# Induction of Stearoyl-CoA 9-Desaturase 1 Protects Human Mesenchymal Stromal Cells Against Palmitic Acid-Induced Lipotoxicity and Inflammation

**DOI:** 10.3389/fendo.2019.00726

**Published:** 2019-10-24

**Authors:** Antoine Dalla Valle, Pascale Vertongen, Delphine Spruyt, Jessica Lechanteur, Valérie Suain, Nathalie Gaspard, Jean-Pierre Brion, Valérie Gangji, Joanne Rasschaert

**Affiliations:** ^1^Laboratory of Bone and Metabolic Biochemistry, Faculty of Medicine, Université libre de Bruxelles, Brussels, Belgium; ^2^Laboratory of Histology, Neuroanatomy and Neuropathology, Faculty of Medicine, ULB Neuroscience Institute (UNI), Université libre de Bruxelles, Brussels, Belgium; ^3^Department of Rheumatology and Physical Medicine, Erasme Hospital, Université libre de Bruxelles, Brussels, Belgium

**Keywords:** human mesenchymal stromal cells (hMSC), fatty acids, lipotoxicity, endoplasmic reticulum stress (ER stress), inflammation, liver X receptor activator, stearoyl-CoA 9-desaturase 1 (SCD1), bone diseases

## Abstract

In bone diseases such as osteonecrosis and osteoporosis, a shift toward a preferential differentiation of mesenchymal stromal cells (MSC) into adipocytes at the expense of the osteoblastic lineage is described, leading to excessive accumulation of adipocytes in the bone marrow of the patients. The influence of cytokines and adipokines secreted by adipocytes on skeletal health is already well-documented but the impact of free fatty acids release on bone cell biology and viability is an emerging concept. We have previously demonstrated that the saturated fatty acid (SFA) palmitate (Palm) is cytotoxic for human MSC (hMSC) and osteoblasts whereas oleate (Ole), a monounsaturated fatty acid (MUFA), has no toxic effect. Moreover, Ole protects cells against lipotoxicity. Our observations led us to propose that the toxicity of the SFA is not correlated to its intracellular accumulation but could rather be related to the intracellular SFA/MUFA ratio, which finally determines the toxic effect of SFA. Therefore, in the present study, we have investigated the potential protective role of the enzyme stearoyl-CoA 9-desaturase 1 (SCD1) against the deleterious effects of Palm. SCD1 is an enzyme responsible for desaturation of SFA to MUFA; its activation could therefore lead to modifications of the intracellular SFA/MUFA ratio. In the present study, we showed that hMSC express SCD1 and liver X receptors (LXRs), transcription factors regulating SCD1 expression. Human MSC treatment with a LXRs agonist triggered SCD1 expression and drastically reduced Palm-induced cell mortality, caspases 3/7 activation, endoplasmic reticulum stress and inflammation. We also observed that, in the presence of Palm, the LXRs agonist provoked lipid droplets formation, augmented the total cellular neutral lipid content but decreased the SFA/MUFA ratio when compared to Palm treatment alone. Addition of an inhibitor of SCD1 activity abrogated the positive effects of the LXRs agonist, suggesting that SCD1 could play a key role in protecting hMSC against lipotoxicity.

## Introduction

Osteoporosis (OP) and non-traumatic osteonecrosis (ON) are bone diseases affecting the quality of life. They are associated with low bone mass and increased fracture risk (1, 2). The prevalence of OP and ON is growing (3) and, although both diseases share some common risk factors (i.e., alcohol abuse and glucocorticoid treatment), their etiology is still not well-understood. It is now established that OP and ON are characterized by a decreased number of bone marrow mesenchymal stromal cells (MSC) and adipocyte accumulation in the bone marrow niche (4–6). Adipocytes release adipokines, cytokines and free fatty acids (FFA) that might influence skeletal cell survival and function and, hence, affect bone remodeling (2, 7–9).

FFA are an essential energy source for various cell types but it is recognized that a chronic exposure to high concentrations of those nutrients may be deleterious for cell function and survival in a process called lipotoxicity. The cellular and molecular mechanisms involved in lipotoxicity are still not completely understood although different processes have been described, including reactive oxygen species production (10), ceramide synthesis (11), and endoplasmic reticulum (ER) stress initiation (12). Lipotoxicity has been widely studied in different cell types including hepatocytes (13), pancreatic β cells (14), podocytes (15), and artery endothelial cells (16) but was poorly investigated in human MSC (hMSC). The role of lipotoxicity in the pathogenesis of bone diseases associated with lipid metabolism abnormalities such as OP and ON is now the subject of growing interest.

In a previous work, we showed that palmitic acid (Palm; C16:0), a saturated fatty acid (SFA), induced apoptosis of hMSC and osteoblasts. Cell death was preceded by activation of endoplasmic reticulum (ER) stress, pro-apoptotic pathways and by induction of a pro-inflammatory state. Addition of oleic acid (Ole; C18:1), a mono-unsaturated fatty acid (MUFA), prevented the deleterious effects of Palm by favoring its channeling and esterification in lipid droplets (LD) (17). We further demonstrated that SaOS-2 cells, a human osteoblastic cell line (18), co-treated with Palm and Ole accumulated higher intracellular amounts of Palm than SaOS-2 cells exposed to Palm alone (17). These observations led us to propose that the toxicity of the SFA is not correlated to its intracellular accumulation but, rather, could be related to the intracellular SFA/MUFA ratio (17).

In order to validate this hypothesis, we have now evaluated the implication of stearoyl-CoA 9-desaturase (SCD1) in hMSC protection against lipotoxicity. SCD1 catalyzes the desaturation of Palm and stearic acid (C18:0) in their monounsaturated counterparts, Cis-9-palmitoleic acid (C16:1) and Ole. Insulin, carbohydrates, SFA, MUFA, polyunsaturated fatty acids (PUFA) and cholesterol (19, 20) modulate expression of SCD1 by activation of different transcription factors such as liver X receptors (LXRs), peroxisome proliferator-activated receptors α, sterol regulatory element-binding proteins and CCAAT-enhancer-binding proteins (C/EBP)α.

LXRs are members of the nuclear hormone receptors superfamily of ligand-activated transcription factors inducing the expression of target genes after ligand binding. Oxysterol, oxidized cholesterol, cholesterol biosynthesis intermediates and glucose are natural ligands of LXRs and synthetic agonists of LXRs like T0901317 and GW3965 are routinely used for *in vitro* experiments (16, 21). LXR has two isoforms: LXRα that is mainly expressed in metabolically active tissues such as liver, intestine, macrophages, and adipose tissue and LXRβ which is ubiquitously expressed (21, 22).

In the present study, we postulate that LXRs activation could protect hMSC from lipotoxicity by modulating SCD1 expression and, consequently, inducing modifications of the intracellular SFA/MUFA ratio. Therefore, we examined the expression and regulation of the two isoforms of LXR in hMSC and we investigated the effect of a synthetic LXRs agonist, T0901317, on gene and protein expression as well as on cell function and survival.

## Materials and Methods

### Isolation, Culture, and Characterization of hMSC

hMSC were obtained from bone marrow aspirated from the posterior iliac crest of healthy volunteers and patient affected by osteonecrosis (all donors were aged ≥18 years). The study was approved by our local institutional ethical committee, Comité d'Ethique hospitalo-facultaire Erasme-ULB (021/406), agreation number by “Ordre des Médecins” OM021. All participants gave their written consent. Bone marrow was diluted 1:0.5 with PBS and overlaid on Ficoll-Paque PREMIUM (GE Healthcare, Diegem, Belgium). Mononuclear cells were isolated after centrifugation and seeded at a density of 5.7 × 10^4^ cells/cm^2^ in DMEM low glucose (1 g/l; Invitrogen, Gent, Belgium) supplemented with 10% FBS (Sigma-Aldrich, Diegem, Belgium), 100 U/ml penicillin and 100 μg/ml streptomycin (Invitrogen, Gent, Belgium). The culture medium was renewed after 4 days of culture and then every 2–3 days until cells reached confluence. hMSC were detached by enzymatic treatment (Trypsin-EDTA; Invitrogen, Gent, Belgium) every week and seeded at a density of 5.7 × 10^3^ cells/cm^2^ until passage 8. In order to confirm the mesenchymal nature of the isolated cells, phenotyping by FACS flow cytometer and differentiation assays were performed (17). SaOS-2 cells, a human osteoblastic cell line (a kind gift from Bone Therapeutics, Gosselies, Belgium) were grown in McCoy's 5A medium (Invitrogen, Gent, Belgium) supplemented with 10% FBS, 100 U/ml penicillin and 100 μg/ml streptomycin. Culture conditions were the same as with hMSC.

### Free Fatty Acid Treatment

Sodium oleate and sodium palmitate (Sigma-Aldrich Diegem, Belgium) were weighted and then solubilized in 90%/10% ethanol/water at 70°C to prepare 50 mM stock solutions. Before use, Palm and Ole were diluted in the appropriate culture medium (see below) containing 1% fatty acid free bovine serum albumin (BSA; Sigma-Aldrich, Diegem, Belgium). Taking into account the stepwise equilibrium model and the respective binding affinities of Palm and Ole for BSA (23) the free concentrations of palm and Ole used in our study are close to the physiological ones (24): it is estimated that in the presence of 0.5 mM total FA and 1% BSA, the free (i.e., unbound to BSA) Palm and Ole concentrations are, respectively, 27 nM and 47 nM (25). For FFA supplementation experiments, cells were cultured for the indicated times in a FA-specific medium composed of DMEM low glucose (hMSC) or McCoy's 5A (SaOS-2 cells) medium supplemented with 1% FBS, 100 U/ml penicillin, 100 μg/ml streptomycin, 1% FFA free BSA (Sigma-Aldrich, Diegem, Belgium) and, when required, diluted Palm and/or Ole (0.25–0.50 mM). As Palm and Ole were solved in ethanol, a similar dilution of the alcohol was added in the control culture condition (e.g., absence of FA).

T0901317 (N-(2,2,2-trifluoro-ethyl)-N-[4-(2,2,2-tri-fluoro-1-hydroxy-1-trifluoromethyl-ethyl)-phenyl]benzene-sulfonamide, Sigma-Aldrich Diegem, Belgium), the LXRs agonist, was solubilized in DMSO and used in pretreatment during 16 or 24 h and for treatment during 16, 24, or 48 h at 1–10,000 nM. CAY 10566 (CAY), the SCD1 inhibitor (3-[4-(2-chloro-5-fluorophenoxy)-1-piperidinyl]-6-(5-methyl-1,3,4-oxadiazol-2-yl)-pyridazine, Santa Cruz Biotechnology, Heidelberg, Germany) was dissolved in DMSO and used at 25 μM during 16, 24, or 48 h of treatment. Diluted DMSO was added in the control condition.

### Determination of Cell Viability

Cells were seeded in 96-well plates at a density of 14.7 × 10^3^ cells/cm^2^ for hMSC, and 20.6 × 10^3^ cells/cm^2^ for SaOS-2 cells. After 3 days, culture media were replaced by the FA-specific medium containing or not increasing concentrations of Palm and/or Ole for the indicated times and concentrations. At the end of the treatment period, the cells were washed and incubated for 15 min with the nuclear binding dyes propidium iodide 10 μg/ml (Sigma-Aldrich Diegem, Belgium) and Hoechst 33342 10 μg/ml (Sigma-Aldrich Diegem, Belgium). Cell viability was examined by inverted microscopy (Axiovision Zeiss, Zaventem, Belgium) with UV excitation (λ excitation/emission: 365/397 nm). Living cells were characterized by their intact nuclei with blue fluorescence, whereas dead cells were depicted by yellow-red fluorescence. In each experimental condition, a minimum of 500 cells were counted.

### Measurement of Caspases-3/7 Activity

Caspases-3/7 activity was determined using the Caspase-Glo® 3/7 assay (Promega, Leiden, The Netherlands). 29.4 × 10^3^ cells/cm^2^ were seeded in 96-well white plates. After 3 days, culture media were replaced by FA-specific medium supplemented with increasing concentrations of Palm, complemented or not with Ole, for the indicated times. At the end of the incubation period, 100 μl of culture medium were replaced by 100 μl of Caspase-Glo reagent resulting in cell lysis and cleavage of the Caspase-Glo® 3/7 substrate by the activated caspases. The luminescent signal generated was detected after 1 h using a Victor2 (PerkinElmer, Zaventem, Belgium).

### Quantification of mRNA Expression by Quantitative Polymerase Chain Reaction (qPCR)

Total RNA was isolated from cells seeded 3 days before treatment in 6-well plates at a density of 10.4 × 10^3^ cells/cm^2^, using AURUM^TM^ kit (Bio-Rad, Nazareth Eke, Belgium) according to the manufacturer's protocol. Total RNA (100 ng) was reverse transcribed in 20 μl using iScript cDNA synthesis kit (Bio-Rad, Nazareth Eke, Belgium). qPCR reactions were performed using a CFX96 thermal system (Bio-Rad, Nazareth Eke, Belgium) in a total reaction volume of 20 μl containing 3 mM MgCl_2_, 0.3 μM (each) forward and reverse primers (Table 1) (Eurogentec, Seraing, Belgium), 10 μl SYBR Green mix (Bio-Rad, Nazareth Eke, Belgium), and 2 μL cDNA. The cycling program was as follow: 95°C for 3 min followed by 40 cycles at 95°C for 30 s and 60°C for 30 sec. Hypoxanthine phosphoribosyltransferase 1 (*HPRT1*) was used as housekeeping gene.

**Table 1:** Primer sequences for real-time PCR.

	**Sequence**	**Product size (bp)**
*BiP (HSPA5*)-For	5′-ACCAATTATCAGCAAACTCTATGGAA-3′	74
*BiP (HSPA5)-*Rev	5′-CATCTTTTTCTGCTGTATCCTCTTCA-3′	
*CHOP (DDIT3)-*For	5′-TGGAAGCCTGGTATGAGGAC-3′	123
*CHOP (DDIT3)-*Rev	5′-AAGCAGGGTCAAGAGTGGTG-3′	
*HPRT1-*For	5′-GGCGTCGTGATTAGTGATGAT-3′	189
*HPRT1-*Rev	5′-CTTGAGCACACAGAGGGCTAC-3′	
*IL6-*For	5′-AGCCACTCACCTCTTCAGAACGAA-3′	122
*IL6-*Rev	5′-CAGTGCCTCTTTGCTGCTTTCACA-3′	
*IL8 (CXCL8)-*For	5′-GGACCACACTGCGCCAACACAG-3′	88
*IL8 (CXCL8)-*Rev	5′-TCCACAACCCTCTGCACCCAGTT-3′	
*LXRα (NR1H3)-*For	5′-CGCACTACATCTGCCACAGT-3′	141
*LXRα (NR1H3)-*Rev	5′-TCAGGCGGATCTGTTCTTCT-3′	
*LXRβ (NR1H2)-*For	5′-CCTCCTGAAGGCATCCACTA-3′	163
*LXRβ (NR1H2)-*Rev	5′-GAACTCGAAGATGGGGTTGA-3′	
*SCD1 (SCD)-*For	5′-TGTTCGTTGCCACTTTCTTG-3′	163
*SCD1 (SCD)-*Rev	5′-TAGTTGTGGAAGCCCTCACC-3′	

### Western Blotting

hMSC (10.4 × 10^3^ cells/cm^2^ in 6-well plates) were treated with FFA and then lyzed with Laemmli buffer (10% glycerol, 0.5 mM DTT, 63 mM Tris-HCl, 1% SDS, pH 6.8) containing a cocktail of proteases and phosphatases inhibitors (Roche Diagnostics, Vilvoorde, Belgium). Proteins were quantified by a paper dye binding assay. Equal protein amounts were separated by SDS-PAGE using 10% polyacrylamide gels. Proteins were transferred on polyvinylidene difluoride (PVDF) membranes Immobilion-P (Millipore, Overijse, Belgium) and immunolabeled overnight at 4°C using SCD1 antibody (rabbit, #2438, lot 2, Cell Signaling Technology, Leiden, The Netherlands). Membranes were then incubated for 1 h at room temperature with the secondary antibody peroxidase-conjugated ECL^TM^ (GE healthcare, Diegem, Belgium). The immune complexes were detected by using the enhanced chemiluminescence method (Western Lightning® *Plus*-ECL, PerkinElmer Inc, Zaventem, Belgium). Band densities were quantified by ImageJ program (NIH, Bethesda, USA). Results were expressed as relative ratio between the protein of interest and β-actine (rabbit, #4967, lot 7, Cell Signaling Technology, Leiden, The Netherlands).

### Detection of Intracellular Lipid Droplets

SaOS-2 cells were seeded in 6 well plates at 20.6 × 10^3^ cells/cm^2^. When required, cells were pretreated with T0901317 (10 μM) during 16 h. After 24 h incubation in the presence or absence of Palm (0.25 mM), T0901317 (10 μM) and/or CAY (25 μM), SaOS-2 cells were washed twice with PBS and fixed with 4% paraformaldehyde for 15 min. The neutral lipids accumulated in intracellular LD were stained by BODIPY™ 493/503 (D3922, Thermofisher, Alost, Belgium) 1 μg/ml for 1 h. Nuclei were stained by Hoechst 33342 10 μg/ml (Sigma-Aldrich, Diegem, Belgium) for 10 min. After coloration, cells were rinsed twice with PBS before microscopic observation, in DMEM without FBS at room temperature using an Axiovert 200M fluorescence microscope and Axiocam MRm (Axiovision Zeiss, Zaventem, Belgium). The images have been merged with Axiovision software and scales have been added with ImageJ program (NIH, Bethesda, USA).

### Ultrastructural Analysis by Electron Microscopy (EM)

SaOS-2 cells were seeded in flask of 75 cm^2^ at a density of 20.6 × 10^3^ cells/cm^2^. Cells were pretreated with or without T0901317 (10 μM) during 16 h and further cultured in control conditions or treated for 24 h with Palm (0.25 mM), T0901317 (10 μM) and/or CAY 10566 (CAY) (25 μM). Cells were then collected and centrifuged at 320 g and the pellets were fixed with 4% (v/v) glutaraldehyde in 0.1 mol/L phosphate buffer (Millonig's buffer) at pH 7.4 for 1 h at RT. After 24 h washing in Millonig's buffer containing 0.5% sucrose (w/v), cells were post-fixed in 2% (w/v) OsO4 for 45 min, dehydrated, and embedded in Epon. Ultrathin sections were counterstained with lead citrate and uranyl acetate and observed with a Zeiss EM 109 at 50 kV (26). Scales have been added on images with ImageJ program (NIH, Bethesda, USA).

### Gas Chromatography Analysis

SaOS-2 cells were seeded in flasks of 75 cm^2^ at a density of 20.6 × 10^3^ cells/cm^2^,and pretreated with or without T0901317 (10 μM) during 16 h. To evaluate the cellular content of FA after 24 h of treatment in the presence or absence of Palm (0.25 mM), T0901317 (10 μM), and/or CAY (25 μM), cells were washed with PBS, scrapped and collected in 1 ml lysis buffer (1% SDS, 60 mM Tris-HCl and 10 mM EDTA). Total lipids were extracted with chloroform/methanol/water (2:2:1) and separated on a solid phase extraction -columns (Bond Elut-NH2, 200 mg, 3 ml, Agilent, Diegem, Belgium) in 3 fractions: free FA, neutral lipids (e.g., mono-, di- and triglycerides and cholesterol esters) and phospholipids. Quantification and characterization of the FA profile of the 3 lipidic fractions were then performed according to Louis et al. (27) on a Agilent 6890 series gas chromatography with a Supelco 24019 column (Sigma-Aldrich, Diegem, Belgium).

### Statistical Analysis

Data are presented as means ± SEM. Values were determined from at least three independent experiments. Statistical analysis was performed by SPSS using Student's *t*-test or one-way ANOVA followed by *t*-test with the Fisher LSD correction for triple comparisons. Differences between groups were considered as statistically significant at *p* < 0.05.

## Results

### LXRs Activation Triggers LXRα Expression in hMSC

T0901317 is a non-steroidal agonist of both isoforms of the transcription factor LXR, inducing the expression of genes under their control (28). To study the effect of LXRs activation, hMSC were pretreated during 16 h with T0901317 (1 nM to 10 μM) before a further 24 h culture period in the absence or presence of 0,25 mM of Palm or Ole.

**Figure 1:**
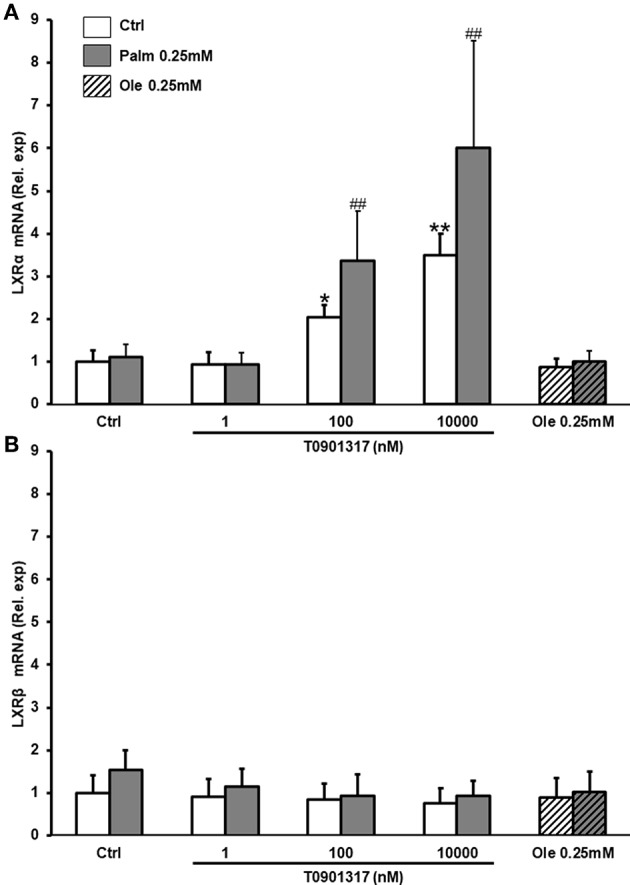
T0901317 increases the expression of LXRα. hMSC were pretreated during 16 h with T0901317 and treated for 24 h with T0901317 in the absence of Palm (white column) or in the presence of Palm 0.25 mM (gray column), Ole 0.25 mM (shaded column). LXRα **(A)**, LXRβ **(B)**, expression were quantified by qPCR using the ΔΔCT method. Values were normalized for HPRT1 expression and are expressed as the ΔΔCT relative to control (Ctrl). Results are mean ± SEM of 9-12 individual experiments. ^*^p < 0.01; ^**^p < 0.001 vs. Ctrl; ^*##*^*p* < 0.001 vs. Palm 0.25 mM.

As previously described in other cell types, T0901317 increased *LXR*α mRNA in a dose-dependent manner in hMSC but neither Palm, nor Ole significantly affected *LXR*α expression (Figure 1A). Neither T0901317, nor Ole (0.25 mM), nor Palm (0.25 mM) modulated the expression of LXRβ, the FFA being tested individually or in combination (Figure 1B).

### LXRs Activation Protects hMSC From Palm Cytotoxicity and Represses Palm-Induced ER Stress and Inflammation

**Figure 2:**
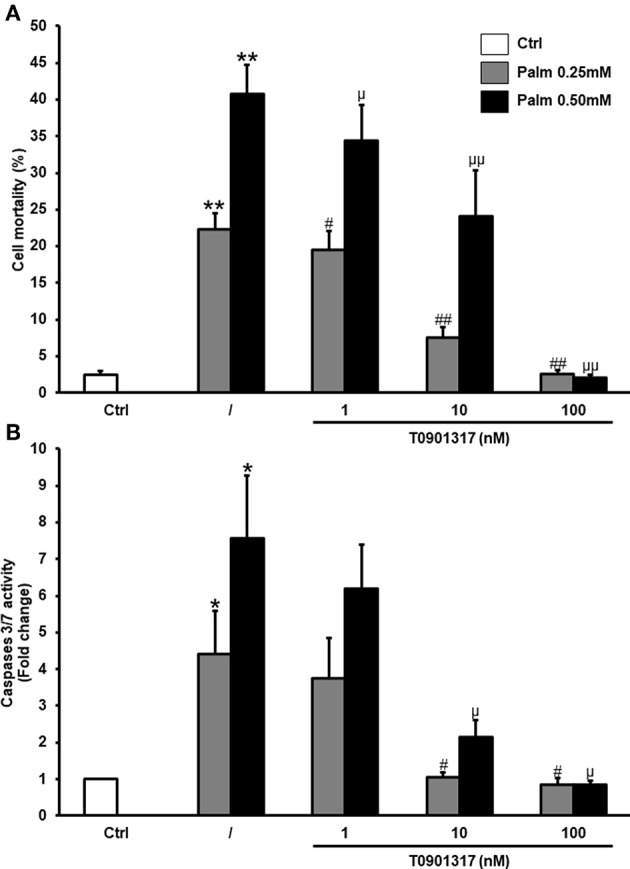
T0901317 decreases Palm-induced cell death. hMSC were pretreated during 16 h with T0901317 and treated 24 h with T0901317 in the absence of Palm (white column) or in the presence of Palm 0.25 mM (gray column) or 0.50 mM (black column). Cell death **(A)** was quantified by nuclear staining with Hoechst and propidium iodide. Values are mean ± SEM of 12–17 individual experiments. Caspases-3/7 activity **(B)** was measured using the Caspases-3/7 Glo assay. Values are expressed relative to Ctrl and are mean ± SEM of 3 individual experiments. ^*^p < 0.05; vs. Ctrl; ^**^p < 0.001; ^#^*p* < 0.05; ^*##*^< 0.001 vs. Palm 0.25 mM; ^μ^*p* < 0.05; ^μμ^ <0.001 vs. Palm 0.50 mM.

As shown before (17), treatment of hMSC with Palm (0.25 mM or 0.50 mM) induced cell death and activated caspases 3/7 (Figures 2A,B). We now demonstrated that addition of T0901317 decreased Palm-triggered cell mortality in a dose-dependent manner. At the highest concentration tested (100 nM), T0901317 abolished cell death and caspases 3/7 activation (Figures 2A,B). T0901317 did not significantly affect cell viability and caspase 3/7 activation (Supplementary Figure 1A).

**Figure 3:**
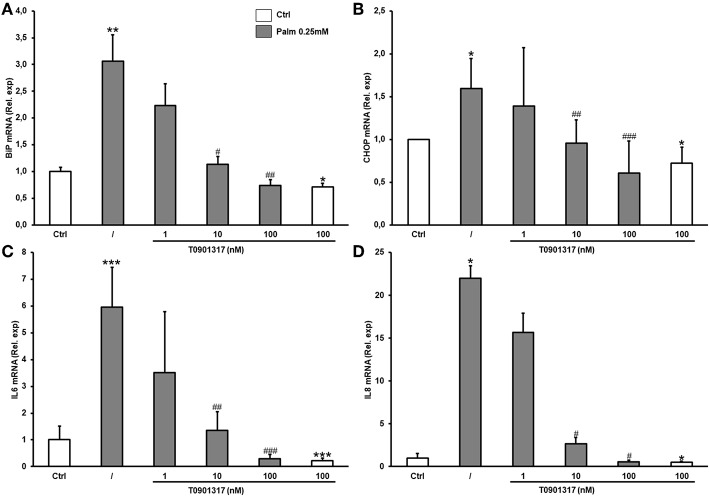
T0901317 counteracts Palm-induced ER stress and inflammation in a dose dependent manner. hMSC were pretreated during 16 h with T0901317 and treated for 24 h with T0901317 in the absence (white column), or in the presence of Palm 0.25 mM (gray column). BiP **(A)** and CHOP **(C)** was used as marker of ER stress. IL6 **(B)**, IL8 **(D)** was used to showed inflammation. Gene expression was quantified by qPCR using the ΔΔCT method. Values were normalized for HPRT1 expression and are expressed as the ΔΔCT compared to control (Ctrl). Results are mean ± SEM of 12 individual experiments. ^*^p < 0.05; ^**^p < 0.01; ^***^p < 0.001 vs. Ctrl; ^#^*p* < 0.05; ^*##*^*p* < 0.01 ^*###*^*p* < 0.001 vs. Palm 0.25 mM.

Ig heavy-chain binding protein (BiP) and C/EBP homologous protein (CHOP) are usually used as ER stress markers. We confirmed that cell treatment with Palm increased *BiP* and *CHOP* mRNA (Figures 3A,B) as well as the expression of the pro-inflammatory cytokines *IL6* and *IL8* (Figures 3C,D). Furthermore, our results revealed that LXRs activation by T0901317 decreased Palm-induced gene expression related to ER stress and cytokines in a dose-dependent manner in hMSC.

### LXRs Activation Increases SCD1 Expression in hMSC

**Figure 4:**
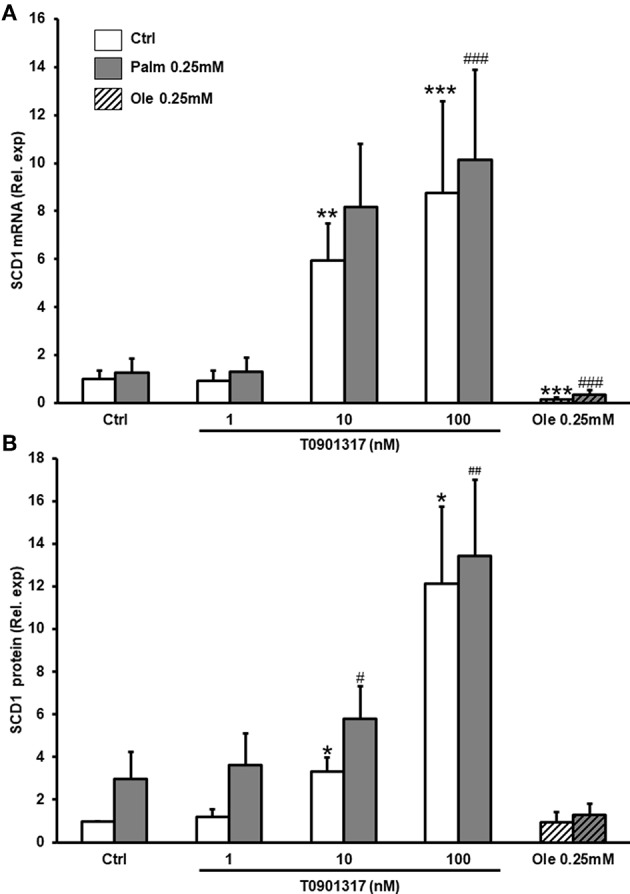
T0901317 increases SCD1 mRNA and protein expression. hMSC were pretreated during 16 h with T0901317 and treated for 24 h with T0901317 in the absence (white column), or presence of Palm 0.25 mM (gray column), Ole 0.25 mM (shaded column). SCD1 mRNA expression **(A)**, was quantified by qPCR using the ΔΔCT method. Values were normalized for HPRT1 expression and are expressed as the ΔΔCT compared to control (Ctrl). Values are mean ± SEM of 12 individual experiments SCD1 protein level **(B)**, was evaluated by Western blotting and normalized for β actine and Ctrl. Values are mean ± SEM of 7 individual experiments. ^*^p < 0.05; ^**^p < 0.01; ^***^p < 0.001 vs. Ctrl; ^#^*p* < 0.05; ^*##*^*p* < 0.01 ^*###*^*p* < 0.001 vs. Palm 0.25 mM.

SCD1 is an enzyme implicated in FFA metabolism by converting SFA into MUFA. Its expression is under the control of several factors including LXRs (20). We observed that T0901317, a LXRs agonist, significantly increased SCD1 expression in a dose-dependent manner at both mRNA and protein levels in hMSC (Figures 4A,B). Palm used alone or in combination with T0901317, did not significantly regulate SCD1 protein expression. Of interest, we observed that Ole, tested alone or in combination with Palm, significantly decreased *SCD1* mRNA (Figure 4A).

### Inhibition of SCD1 Activity Abolishes the Protective Action of the LXRs Agonist in hMSC

**Figure 5:**
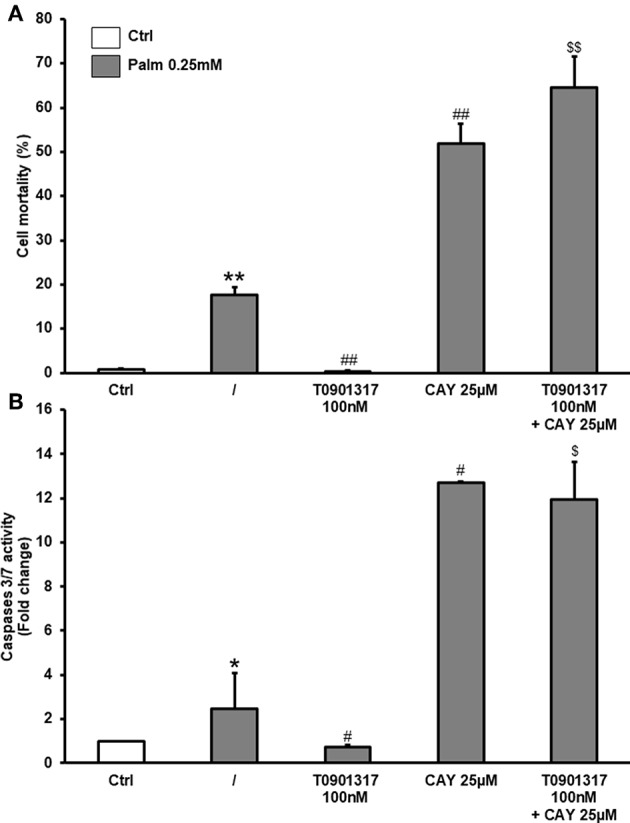
CAY suppresses T0901317 effect on cell viability and caspases 3/7 activation. hMSC were pretreated during 16 h with T0901317 and treated 48 h with T0901317, CAY 25 μM in the absence (white column) or presence of Palm 0.25 mM (gray column). Cell death **(A)** was quantified by nuclear staining with Hoechst and propidium iodide. Values are mean ± SEM of 4 individual experiments. Caspases-3/7 activity **(B)** was measured using the Caspases-3/7 Glo assay. Values are expressed relative to Ctrl and are mean ± SEM of 3 individual experiments. ^*^p < 0.05; ^**^p < 0.01 vs. Ctrl; ^#^*p* < 0.05; ^*##*^*p*< 0.01 vs. Palm 0.25 mM; ^*$*^*p* < 0.05; ^*$$*^*p* < 0.01 vs. Palm 0.25 mM + T0901317 100 nM.

CAY 10566 (CAY) is a potent and selective inhibitor of SCD1 activity, thus preventing the conversion of SFA into MUFA (29). CAY did not significantly affected cell death or caspase activity (Supplementary Figure 1B). In the presence of Palm 0.25 mM, inhibition of SCD1 by CAY significantly magnified the toxicity of the SFA, increasing both cell death and caspases 3/7 activation. Furthermore, our results demonstrated that inhibition of SCD1 activity abolished the protective action of the LXRs agonist T0901317 on Palm-triggered cell death and caspases 3/7 activation (Figure 5).

### Activation of LXRs Increases FFA Incorporation in Lipid Droplets in SaOS-2 Cells

We further evaluated the impact of LXRs activation and SCD1 inhibition on ER morphology in Palm-treated cells. Cell imaging by EM and FA quantification and analysis require large amounts of biological material. As hMSC are a rare and precious resource, SaOS-2 cells, a human osteoblastic cell line, were used to perform EM as we previously demonstrated that those cells are highly sensitive to lipotoxicity, showing a greater decrease of cell viability and a stronger activation of caspases 3/7 and ER stress in response to Palm than hMSC and hMSC-derived osteoblasts (17). The present results documented that LXRs activation increased SCD1 expression and viability in SaOS-2 cells (Supplementary Figures 2A,B), as observed in hMSC (Figures 2A, 4A).

**Figure 6:**
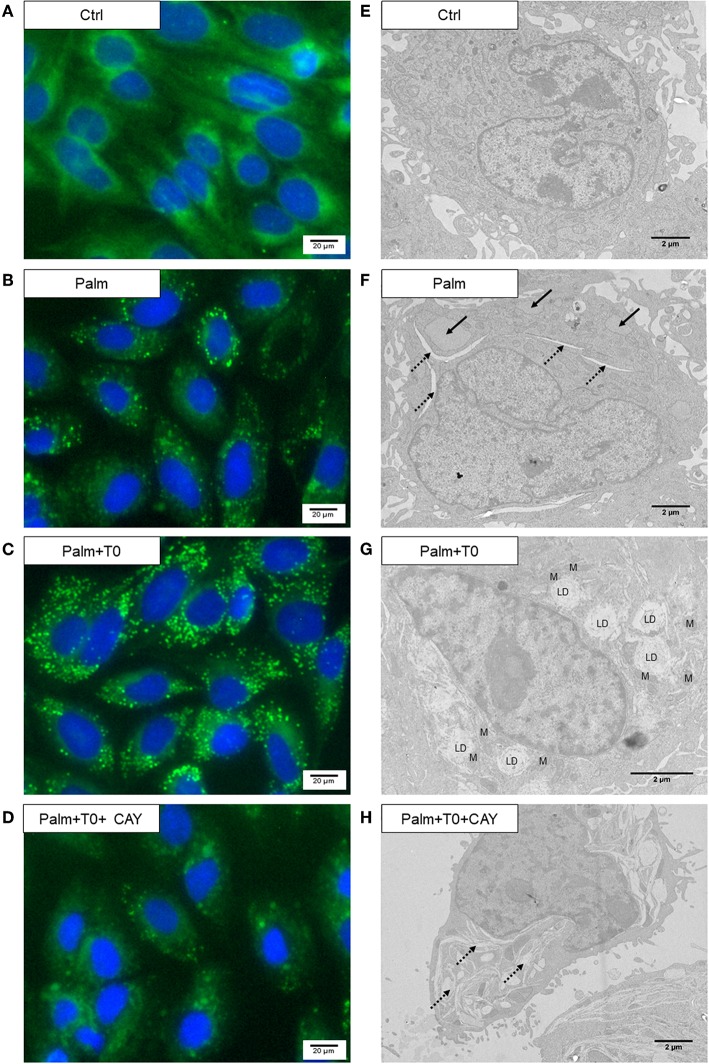
T0901317 induces LD formation in the presence of Palm and counteracts Palm-induced ER disturbance. SaOS-2 cells were pretreated during 24 h with 10 μM T0901317 (T0) and treated for 16 h with T0901317 10 μM, CAY 25 μM in absence, or in the presence of Palm 0.25 mM. Bodipy staining of lipidic vacuoles, microscopic observations were performed with a 20 fold magnification lenses **(A–D)**. Electron micrographs **(E–H)**. Arrow, inflated ER; discontinued arrow, angular ER; LD, lipid droplet; M, mitochondria.

Lipid droplets (LD) were not detected in SaOS-2 cells cultured in control condition (Figures 6A,E), whereas, as shown in Figure 6B, Palm-treated SaOS-2 cells displayed bodipy signal and we observed by EM that the ER was disrupted and inflated or angular (Figure 6F). Bodipy fluorescence markedly increased in SaOS-2 cells co-treated with Palm and T0901317 (10 μM) (Figure 6C). Moreover, we noticed the presence of numerous large LD surrounding the nucleus and often located in contact with, or close to, mitochondria (Figure 6G); the ER structure appeared unaltered in most of the cells. Addition of CAY, the SCD1 inhibitor, to Palm and T0901317 suppressed LD formation, severely damaged the ER (Figures 6D–H) and induced cell death (Supplementaury Figure 2B).

### LXRs Activation Abolishes Palm-Induced Modifications of the SFA/MUFA Ratio in SaOS-2 Cells

We further characterized and quantified the fatty acids (FA) present in the FFA, neutral lipids (NL) and phospholipids (PL) fractions obtained from SaOS-2 cell lysates cultured in control conditions or in the presence of Palm, Palm and T0901317, and/or CAY. The FA profile (i.e., SFA, MUFA, and PUFA contents) of the FFA fraction was unaffected by the different culture conditions (data not shown).

**Figure 7:**
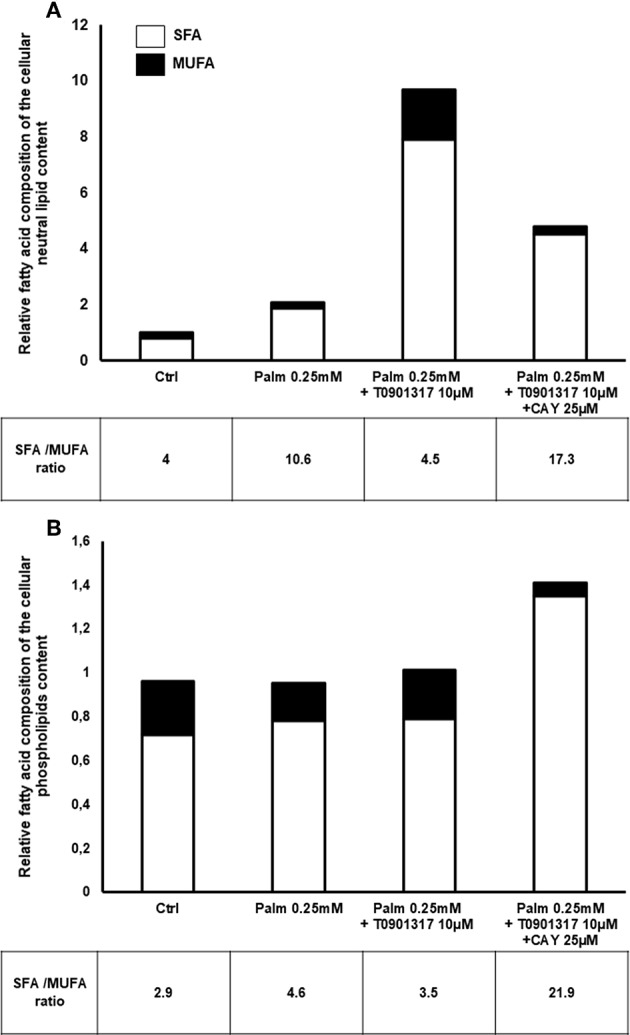
Relative FA content in SFA and MUFA. Normalized FA composition of SaOS-2 cells extract regroup in 2 classes: SFA and MUFA. Values are expressed relative to Ctrl. SaOS-2 cells were pretreated during 24 h with T0901317 and treated 16 h with T0901317 10 μM, CAY 25 μM in absence, or in the presence of Palm 0.25 mM. The fat extract is separated in 3 fractions: NL **(A)**, PL **(B)** and FFA (data not shown). Values are mean ± SEM from 4 to 7 individual experiments.

When cells were exposed to Palm, we observed an important accumulation of SFA in the NL fraction whereas the MUFA content was slightly decreased (Figure 7A). Of note, myristic acid (C14:0) augmented but its total amount remained smaller than Palm (Table 2). Taken together, these modifications led to an increase of the SFA/MUFA ratio from 4 to 11 in SaOS-2 cells exposed to Palm.

**Table 2:** Modifications of SFA and MUFA content in SaOs-2 cells treated with Palm, an agonist of LXRs and/or a SCD1 inhibitor.

**Fatty acid**	**Ctrl**	**Palm 0.25 mM**	**Palm 0.25 mM + T0901317 10 μM**	**Palm 0.25 mM + T0901317 10 μM + CAY 25 μM**
**NEUTRAL LIPIDS**				
C14:0	0.05 ± 0.03	0.30 ± 0.12	1.89 ± 0.31^##^	0.85 ± 0.20^$$$^
C16:0	3.59 ± 0.11	14.66 ± 0.61^**^	76.13 ± 9.95^###^	39.87 ± 5.91^$$$^
C18:0	5.04 ± 0.30	5.63 ± 0.38	7.69 ± 0.62	8.36 ± 0.89
**Total SFA**	**8.67** **±** **0.38**	**20.58** **±** 0.58^***^	**85.99** **±** **10.83**^###^	**49.08** **±** **6.51^$$$^**
C16:1cis9	ND	0.30 ± 0.11^*^	7.81 ± 1.07^###^	0.28 ± 0.11^$$$^
C18:1cis9	1.08 ± 0.27	1.18 ± 0.07	5.30 ± 0.85^##^	1.58 ± 0.22^$$^
C18:1cis11	1.09 ± 0.41	0.44 ± 0.28	5.67 ± 0.83^###^	0.95 ± 0.48^$$$^
**Total MUFA**	**2.17** **±** **0.61**	**1.93** **±** **0.37**	**19.09** **±** **2.75**^###^	**2.84** **±** **0.72^$$$^**
**PHOSPHOLIPIDS**				
C14:0	0.91 ± 0.21	0.56 ± 0.18^*^	0.40 ± 0.16^#^	1.07 ± 0.32^$^
C16:0	17.04 ± 2.55	20.08 ± 4.01	19.85 ± 3.90	36.11 ± 7.89^$^
C18:0	5.90 ± 0.77	5.38 ± 0.71	5.99 ± 0.88	7.68 ± 1.56
**Total SFA**	**23.85** **±** **3.37**	**26.02** **±** **4.86**	**26.24** **±** **4.85^#^**	**44.86** **±** **9.67^$^**
C16:1cis9	0.71 ± 0.09	1.10 ± 0.19	2.06 ± 0.46^#^	0.12 ± 0.12^$^
C18:1cis9	4.86 ± 0.66	3.06 ± 0.47^*^	3.42 ± 0.62	1.29 ± 0.66^S^
C18:1cis11	2.52 ± 0.30	1.42 ± 0.19^*^	1.85 ± 0.30	0.62 ± 0.38^$^
**Total MUFA**	**8.20** **±** **1.07**	**5.66** **±** **0.88**	**7.40** **±** **1.41^#^**	**2.05** **±** **1.13^$^**

*Main FA extracted in SaOS-2 cells. SaOS-2 cells were pretreated during 24 h with T0901317 and treated 16 h with Palm 0.25 mM in the presence or absence of T0901317 10 μM and CAY 25 μM. Values are mean ± SEM of 4–7 individual experiments and are expressed in ng of fatty acid per μg of cell protein*.

* p <0.05;

** p <0.01 vs. Ctrl;

*** p <0.001;

# p <0.05;

## p <0.01;

### p <0.001 vs. Palm 0.25 mM;

$ p <0.05;

$$ p <0.01;

$$$ *p <0.001 vs. Palm 0.25 mM + T0901317 100 nM*.

As shown in Table 2, activation of LXRs by T0901317 in the presence of Palm induced a massive cellular accumulation of NL (10 times the amount measured in the control condition). As both SFA (from 9 to 86 ng/μg cell protein) and MUFA (from 2 to 19 ng/μg cell protein) increased, LXRs activation restored the SFA/MUFA ratio close to its control value (Figure 7A). Of note, Palm represented 65% of the total SFA.

In Palm + T0901317 treated cells, inhibition of SCD1 by CAY decreased twice the NL fraction, as compared to Palm + T0901317 treated cells. As total SFA and MUFA were decreased by 40 and 85%, respectively, the SFA/MUFA ratio increased to 17.3, principally due to modifications of Palm and of Cis-9-palmitoleic acid, Ole and Cis-11-vaccenic acid contents (Table 2).

In the PL fraction (Figure 7B), Palm or Palm + T0901317 treatments did not significantly affect SFA and MUFA content. However, addition of CAY to Palm and T0901317 doubled the SFA content while decreasing 4 times the quantity of MUFA. The SFA/MUFA ratio was thus only significantly affected by the concomitant presence of Palm, T0901317 and CAY in the PL fraction. Of note, despite the fact that we observed that the total lipid content was not significantly modified by Palm, the SFA/MUFA ratio was enhanced by around 50% as Palm content increased while Ole and Cis-11-vaccenic acid amounts decreased (Figure 7B, Table 2). Conversely, addition of T0901317 to Palm reduced the SFA/MUFA ratio, although in a non-significant manner, compared to Palm alone treatment (Figure 7B).

Of note, as expected, in both the NL and PL fractions, Palm slightly raised the quantity of Cis-9-palmitoleic acid whereas addition of T0901317 largely increased it (Table 2).

The objective of the present work is to evaluate the hypothesis that Palm lipotoxicity is prevented by decreasing the SFA/MUFA ratio in hMSC. To reach this purpose, cells were treated with a LXRs agonist in order to trigger SCD1 expression. SCD1 is an enzyme located in the ER membrane where it catalyzes the desaturation of SFA in their monounsaturated counterparts; its expression is upregulated by nutrients such as glucose, fructose, SFA and by LXR activation (30). Due to the very short half-life (3–4 h) of SCD1 protein, the activity of the enzyme is easily controlled via regulation of its expression (31).

## Discussion

LXRs are mainly known for their regulatory function on lipid and carbohydrate metabolism by oxysterols, the main natural LXRs ligands (32, 33). Indeed, LXRs regulate expression of genes involved in cholesterol metabolism and efflux. In the liver, LXRs induce the expression of several enzymes, including SCD1 (34). Oxysterols were implicated in osteoblastic and adipogenic differentiation of murine MSC, although their influence is still under debate. In mouse bone marrow-derived MSC, it was reported that activation of LXRs inhibits sonic hedgehog-induced osteoblastic differentiation (35). At the opposite, oxysterols were shown to promote osteoblastic differentiation by activation of both the hedgehog (36) and the LXRs signaling pathways (37) while they reduce adipogenic differentiation in a mouse multipotent bone marrow stromal cell line (36). Moreover, it was documented that oxysterol 20(S)-Hydroxycholesterol stimulates osteogenic differentiation by inducing notch target gene expression (37). Interestingly the overexpression of LXRα has an inhibitory effect on adipocyte differentiation (38), and LXRs activation was shown to decrease osteoclastogenesis and to trigger osteoclast apoptosis (39), thus reducing *in vitro* bone resorption (40). In a co-culture osteoporosis model of mouse osteoblasts and osteoclasts, LXRs activation counteracts osteoclastogenesis via inhibition of receptor activator of nuclear factor kappa-B ligand (RANKL) production by osteoblasts. In this model, activation of LXRs restores a physiological RANKL/osteoprotegerin ratio, promoting bone homeostasis (41). Up to now, the impact of LXRs activation on the biology of human MSC has been poorly investigated. In a particular cell sub-population, the multilineage-differentiating stress enduring (Muse) cells, LXRs activation has been linked to stem cell self-renewal and immunomodulation (42). The presence of LXRs-responsive elements in the LXRα promoter was previously documented, allowing regulation of LXRα expression by its own ligand in different cell types (43). In the present study, we confirm that hMSC expressed both LXR isoforms and we demonstrate that LXRs activation increases LXRα expression [the isoform particularly implicated in the control of lipid biosynthesis (22)] without affecting LXRβ expression. We further reveal for the first time that LXRs activation totally counteracts the deleterious effects of Palm in hMSC. LXRs were recently described as a connection between lipid metabolism and immune response (44). Indeed, in macrophages, LXRs activation counteracts the effects of LPS on expression of cytokines such as IL6 and IL1β and of the chemokine monocyte chemoattractant protein-1 (MCP-1), probably through modulation of NF-kB activity (45). In hMSC, our results show that LXRs activation decreases the inflammatory process triggered by Palm as IL6 and IL8 expression are significantly reduced. These observations are in line with the results of Wang et al. who demonstrated that activation of LXRs by artificial ligand decreases inflammation via toll-like receptor 4 (TLR4)/NF-κB and Keap-1/Nrf-2 pathways in adipose-derived mouse MSC (46). In macrophages, TLR4 activation reduces LXRs activation and decreases LXRs target gene expression after bacterial infection (47), suggesting a crosstalk between the two signaling pathways. Of interest, SFA are endogenous ligands of TLR4 and we have previously showed that Palm activates NF-kB and increases TLR4 expression as well as IL6 and IL8 secretion in hMSC, suggesting that TLR pathway might be embroiled in Palm-induced lipotoxicity in hMSC (17).

LXRs agonists such as SFA are known to upregulate SCD1 expression while MUFA and PUFA decrease SCD1 transcription in human aortic smooth muscle cells and mouse hepatocytes (30, 31). In the present work, we demonstrate for the first time a dose dependent effect of T0901317, the LXRs agonist on both SCD1 mRNA and protein expression in hMSC. As expected, Ole decreases SCD1 transcription. However, we could not show a significant effect of Palm on SCD1 mRNA and protein expression but this could be related to the high variability of SCD1 expression between subjects (Supplementary Figure 3), as previously documented (48). Our observations are in fair agreement with the literature as Karaskov et al., showed that medium-chain SFA, and particularly lauric acid (C12:0), bind and activate LXRα whereas long-chain SFA have no effect (49). Moreover, Bedi et al. observed that Palm binds to but does not activate LXRα in LXRα-transfected COS-7 cells (50).

The molecular mechanisms involved in SFA-induced lipotoxicity are still not fully understood. Various mechanisms were described (10–12), some are common to all cell types while others are specific of the cell type examined. SFA may provoke cell death by induction of an ER stress as Palm accumulation in the ER induces its engorgement, leading to disruption of the ER homeostasis and activation of the unfolded protein response (UPR) (51). UPR initially acts as a pro-survival response by triggering distinct mechanisms to overcome the ER overload: decreased protein translation, increased ER chaperone synthesis and misfolded protein clearance. If UPR does not successfully manage the ER engorgement, the response switches to activation of a pro-apoptotic pathway and, finally, leads to cell death (13, 41). In hMSC, we previously demonstrated that Palm treatment activates UPR by increasing expression of BiP, an ER chaperone and CHOP, an UPR-related protein inducing cell death. Activation of UPR is concomitant with caspases 3/7 activation and cell death (17). We now demonstrate that the LXRs agonist T090137 counteracts Palm activation of the UPR pathway and of caspases 3/7, thus promoting hMSC survival. All these effects are likely mediated by activation of SCD1 expression since inhibition of SCD1 activity abrogates the beneficial impacts of T090137.

In INS-1E cells, a rat pancreatic β cell line, downregulation of SCD1 expression enhances ER stress and apoptosis (52) whereas increasing SCD1 expression prevents lipotoxicity by favoring the incorporation of Palm in LD (53) These observations are in line with the present work, as we showed that the LXRs agonist T0901317 triggers SCD1 expression, LD formation and protects hMSC from Palm-induced toxicity. To further characterize the effects of Palm on cell organelles by EM, we used SaOS-2 cells, a human osteoblastic cell line, as susceptible to lipotoxicity than hMSC and hMSC-derived osteoblasts (17). Our results establish that Palm treatment distends the ER and induces ER membrane rupture. SaOS-2 cells co-treated with Palm and the LXRs agonist display LD formation and present less ER abnormalities such as inflated, angular or disrupted ER and cell viability is preserved. Lastly, we show that addition of an inhibitor of SCD1 activity to Palm and T0901317 suppress LD formation, provokes substantial damage to the whole cytoplasm and magnifies cell death, demonstrating the key role of SCD1 in the anti-lipotoxicity action of the LXRs agonist.

To further characterize the molecular mechanisms involved in the beneficial action of LXRs activation, we used gas chromatography to determinate the changes in nature and amount of the FA constituting LD and membrane in cells cultured in our different experimental conditions. Cell lipid extracts were separated into three fractions: (1) neutral lipids (NL) that are essentially composed of triglycerides, (2) phospholipids (PL), containing the membrane phospholipids, and (3) FFA, the non-esterified fatty acids. We observed that the lipid composition of this last fraction is unaffected by the different treatments tested (data not shown), as previously stated by Ariyama et al. in Hela cells (54).

In the NL fraction, Palm treatment increases the total lipid amount as well as the SFA/MUFA ratio, which is linked to enhanced cell death. Addition of the LXRs agonist to Palm drastically augments the total amount of NL, particularly the MUFA part, resulting in a decreased SFA/MUFA ratio that is associated with enhanced LD formation, as identified by EM. Upregulation of SCD1 protein synthesis by LXRs activation protects cells from lipotoxicity probably by promoting conversion of Palm to palmitoleic acid and, therefore, favoring its further integration in triglycerides and storage in LD. Indeed, it is known that MUFA are more easily added to diacylglycerol than SFA by the triglyceride-forming enzyme diacylglycerol O-acyltransferase (55). Inhibition of SCD1 activity drastically decreases the MUFA content, leading to a rise of the SFA/MUFA ratio and increased cell mortality. Altogether, these results corroborate the hypothesis that the beneficial action of the LXRs agonist is mediated by SCD1 increased expression and activity. As ER stress is also induced by alteration of the ER membrane fluidity (14, 54, 56), we hypothesized that in Palm-treated hMSC, an increase of saturated PL content could favor stiffening of the membranes and, therefore, participate to ER stress and cell mortality. Indeed, Chamulitrat et al. observed a 2.12 fold increase of the SFA/MUFA ratio in PL concomitant to caspases 3/7 activation when mouse hepatocytes are treated with Palm (56). In our work, even if Palm treatment has no significant effect on the SFA/MUFA ratio in the PL fraction, we observe nevertheless a 1.59 fold increase. Such a rise is weak but was reproducible; improving the experimental protocol by purification of ER membranes could probably permit to reach significant statistical results but this would require a huge number of cells to be performed. Furthermore, our results are concordant with literature and with our EM observations, supporting the idea that the degree of saturation of PL is embroiled in ER stress and cell death (14, 56).

Modifications of the PL membrane SFA/MUFA ratio affects membrane properties by several mechanisms and a membrane enriched in saturated PL shows perturbation of protein activity. Li et al. demonstrated on RAW 264.7 macrophages that saturated PL inhibit the activity of the sarco(endo)plasmic reticulum calcium ATPase-2b (SERCA2b), a calcium pump (57), leading to ER Ca^2+^ depletion and ER stress. In addition, increasing PL membrane saturation triggers the recruitment of cellular Src kinase to the membrane, activates c-Jun N-terminal kinases (JNK) signaling pathway and so, potentially induces inflammation (58). In hMSC, Palm causes inflammation but we have not observed modifications of JNK phosphorylation (data not shown). Palm may also affect membrane properties simply by modifying its hydrophobic nature as Palm aggregates within the membranes and thus increases membrane permeability (59). While addition of the LXRs agonist to Palm abolishes cell death, we have only observed a weak decrease of the SFA/MUFA ratio in the PL fraction (Figure 7B), mainly due to a significant rise (>25 fold) of Cis-9-palmitoleic acid (Table 2). In hMSC, such an increase could indeed partially restore membrane fluidity and thus contributes to preservation of cell viability, as mentioned in the literature (60).

Taken as a whole, our results suggest that lipotoxicity is not related to the accumulation of intracellular Palm but rather to the SFA/MUFA ratio in NL and PL fractions. Indeed, hMSC co-treated with Palm and the LXRs agonist have the largest amount of Palm in the NL fraction, a SFA/MUFA ratio similar to the control condition and do not suffer from lipotoxicity. In contrast, cells treated with Palm alone or in combination with the LXR agonist and the inhibitor of SCD1 contain less Palm in the NL fraction, present a high SFA/MUFA ratio and increased cell death.

ON and OP are bone diseases sharing common similarities such as bone marrow adipocyte accumulation as well as blood increase in triglycerides, high density lipoprotein (HDL) and cholesterol concentrations (61, 62). In a previous work, we have demonstrated that the bone marrow microenvironment of ON patients is enriched in SFA when compared to healthy subjects (9). Moreover, hMSC isolated from ON patients (ON_MSC_) are more sensitive to lipotoxicity than hMSC obtained from healthy volunteers (HV_MSC_). These observations were associated with an inverse regulation of SCD1 expression by Palm: Palm triggered SCD1 expression in HV_MSC_ whereas it reduced it in ON_MSC_ (9). Moreover, the basal expression level of carnitine palmitoyltransferase I, the limiting enzyme of FA β-oxidation, was 2 fold lower in ON_MSC_ than in HV_MSC_, indicating that lipid degradation is also affected in ON_MSC_ (9). Of note, dysfunctions of the NF-kB, TLR and TNF pathways implicating LXRs were recently highlighted in ON and OP patients (44, 63, 64). Altogether our observations suggest an important role for SCD1 in the prevention of lipotoxicity in hMSC.

Of note, dysfunctions of the NF-kB, TLR and TNF pathways implicating LXRs were recently highlighted in ON and OP patients (44, 63, 64).

In animal models, LXRs agonists are tested to treat hyperlipidemia (65) and atherosclerosis (66). Activation of LXRs has also been studied to treat diabetes. In an *in vitro* human hyperinsulinemia-induced insulin resistance model, activation of LXRs restores insulin sensitivity and decreases inflammatory phenotype. In such a model, addition of T0901317 restores insulin-stimulated glucose up-take, fatty acid synthase expression and counteracts metabolic disorder by decreasing secretion of the pro-inflammatory cytokine IL6 (67). Due to their ability to counteract lipotoxicity and their putative positive action on bone formation via decreased adipocyte/osteoclast differentiation and favoring osteoblast differentiation (36–38), LXRs agonists appear to be hopeful treatments for bone diseases associated with lipid metabolic disorders like ON and OP. Unfortunately artificial LXRs ligands like T0901317 or GW3965 increase serum triglyceride levels and exacerbate steatosis (68). Use of a selective LXRβ agonist could eventually maintain the beneficial effects as the triggering of SCD1 expression and the anti-inflammatory effect and suppress the impact on hepatic lipogenesis [for review (69)]. Another strategy proposed to avoid deleterious effects on liver is the use of tissue-selective drugs carrier (69). Instead of increasing SCD1 expression via LXRs activation, it would also be conceivable to increase lifespan of the protein by inhibiting the proteasome or by stabilizing the protein through phosphorylation of Y55 (70, 71).

In conclusion, our work demonstrates the essential role of SCD1 as a protective agent against lipotoxicity in bone marrow hMSC. Of interest, a recent study shows modification of SCD1 methylation at the menopausal age (72). Hypermethylation may impact enzyme expression and therefore, activity, and it is well-known that menopausal women are frequently affected by osteoporosis.

Our work also highlights the importance of maintaining a low intracellular SFA/MUFA ratio to preserve cell viability and validates our hypothesis that the SFA/MUFA ratio rather than Palm accumulation is responsible for cell death. This proposal is further supported by the work of Li et al., showing by NMR spectroscopy studies that bone marrow adipose tissue of ON patients is enriched in SFA and depleted of MUFA, leading to an increase of the SFA/MUFA ratio, when compared to controls subjects. Moreover, they correlated a high SFA/MUFA ratio with a lowest bone mineral density (73). In this work we performed *in vitro* studies on primary cultures of hMSC. Further *in vivo* studies, using animal models of OP and ON, are needed to better understand the physiological roles of SCD1 and LXRs activation in bone remodeling.

Altogether, our observations support future investigations for the use of LXRs agonists as potential therapeutic tools for bone diseases associated with lipid metabolism dysfunction.

## Data Availability Statement

The datasets generated for this study are available on request to the corresponding author.

## Author Contributions

AD conceived and designed the study. AD, PV, and JR analyzed the data and wrote the paper. PV, DS, J-PB, VG, and JR critically reviewed the results. JL, NG, and VS made substantial contributions to acquisition of data. All authors contributed to revise the manuscript.

### Conflict of Interest

The authors declare that the research was conducted in the absence of any commercial or financial relationships that could be construed as a potential conflict of interest.

## Abbreviations

CAY: CAY 10566
C/EBPα: CCAAT-enhancer-binding proteins
EM: electronic microscopy
ER: endoplasmic reticulum
FA: fatty acids
hMSC: human mesenchymal stromal cells
HV_MSC_: hMSC obtained from healthy volunteers
LD: lipid droplet
LXRs: liver X receptors
MCP1: monocyte chemoattractant protein-1
MSC: mesenchymal stromal cells
MUFA: monounsaturated fatty acid
NL: neutral lipids
Ole: oleic acid
ON: non-traumatic osteonecrosis
ON_MSC_: hMSC isolated from ON patients
OP: osteoporosis
Palm: palmitic acid
PL: phospholipids
PUFA: polyunsaturated fatty acids
RANKL: nuclear factor kappa-B ligand
SFA: saturated fatty acid
SCD1: stearoyl-CoA 9-desaturase 1
TLR4: toll-like receptor 4
UPR: unfolded protein response.

## References

[1] Motyl KJ Guntur AR Carvalho AL Rosen CJ. Energy metabolism of bone. Toxicol Pathol. (2017) 45:887–93. 10.1177/0192623317737065 29096593 PMC5777524

[2] Gangji V Soyfoo MS Heuschling A Afzali V Moreno-Reyes R Rasschaert J. Non traumatic osteonecrosis of the femoral head is associated with low bone mass. Bone. (2018) 107:88–92. 10.1016/j.bone.2017.11.005 29154968

[3] Mont MA Cherian JJ Sierra RJ Jones LC Lieberman JR. Nontraumatic osteonecrosis of the femoral head: where do we stand today? A ten-year update. J Bone Joint Surg Am. (2015) 97:1604–27. 10.2106/JBJS.O.00071 26446969

[4] Hernigou P Beaujean F Lambotte JC. Decrease in the mesenchymal stem-cell pool in the proximal femur in corticosteroid-induced osteonecrosis. J Bone Joint Surg Br. (1999) 81:349–55. 10.1302/0301-620X.81B2.8818 10204950

[5] Sheng H Sheng CJ Cheng XY Zhan G Lee KM Leung KS. Pathomorphological changes of bone marrow adipocytes in process of steroid-associated osteonecrosis. Int J Clin Exp Pathol. (2013) 6:1046–50. 23696921 PMC3657356

[6] Hardouin P Rharass T Lucas S Bone marrow adipose tissue: to be or not to be a typical adipose tissue? Front Endocrinol. (2016) 7:85 10.3389/fendo.2016.00085 PMC4928601 27445987

[7] Rosen CJ Bouxsein ML. Mechanisms of disease: is osteoporosis the obesity of bone? Nat Clin Pract Rheumatol. (2006) 2:35–43. 10.1038/ncprheum0070 16932650

[8] Drosatos-Tampakaki Z Drosatos K Siegelin Y Gong S Khan S Van Dyke T. Palmitic acid and DGAT1 deficiency enhance osteoclastogenesis, while oleic acid-induced triglyceride formation prevents it. J Bone Miner Res. (2014) 29:1183–95. 10.1002/jbmr.2150 24272998 PMC4945760

[9] Gillet C Dalla Valle A Gaspard N Spruyt D Vertongen P Lechanteur J. Osteonecrosis of the femoral head: lipotoxicity exacerbation in msc and modifications of the bone marrow fluid. EndocrinologyJN. (2017) 158:490–502. 10.1210/en.2016-1687 28359085

[10] Listenberger LL Ory DS Schaffer J E. Palmitate-induced apoptosis can occur through a ceramide-independent pathway^*^. J Biol Chem. (2001) 276:14890–5. 10.1074/jbc.M010286200 11278654

[11] Kolesnick RN Krönke M. Regulation of ceramide production and apoptosis. Annu Rev Physiol. (1998) 60:643–65. 10.1146/annurev.physiol.60.1.643 9558480

[12] Boslem E Weir JM MacIntosh G Sue N Cantley J Meikle PJ. Alteration of endoplasmic reticulum lipid rafts contributes to lipotoxicity in pancreatic β-cells. J Biol Chem. (2013) 288:26569–82. 10.1074/jbc.M113.489310 23897822 PMC3772204

[13] Zámbó V Simon-Szabó L Szelényi P Kereszturi E Bánhegyi G Csala M. Lipotoxicity in the liver. World J Hepatol. (2013) 5:550–7. 10.4254/wjh.v5.i10.550 24179614 PMC3812457

[14] Moffitt JH Fielding BA Evershed R Berstan R Currie JM Clark A. Adverse physicochemical properties of tripalmitin in beta cells lead to morphological changes and lipotoxicity *in vitro*. Diabetologia. (2005) 48:1819–29. 10.1007/s00125-005-1861-9 16094531

[15] Sieber J Weins A Kampe K Gruber S Lindenmeyer MT Cohen CD. Susceptibility of podocytes to palmitic acid is regulated by stearoyl-CoA desaturases 1 and 2. Am J Pathol. (2013) 183:735–44. 10.1016/j.ajpath.2013.05.023 23867797 PMC3763774

[16] Peter A Weigert C Staiger H Rittig K Cegan A Lutz P. Induction of stearoyl-CoA desaturase protects human arterial endothelial cells against lipotoxicity. Am. J Physiol. (2008) 339–49. 10.1152/ajpendo.00022.2008 18523127

[17] Gillet C Spruyt D Rigutto S Dalla Valle A Berlier J Louis C. Oleate abrogates palmitate-induced lipotoxicity and proinflammatory response in human bone marrow-derived mesenchymal stem cells and osteoblastic cells. Endocrinology. (2015) 156:4081–93. 10.1210/en.2015-1303 26327577

[18] Murray E Provvedini D Curran D Catherwood B Sussman H Manolagas S. Characterization of a human osteoblastic osteosarcoma cell line (SAOS-2) with high bone alkaline phosphatase activity. J Bone Miner Res. (2009) 2:231–8. 10.1002/jbmr.5650020310 2843003

[19] Waters KM Miller CW Ntambi JM. Localization of a polyunsaturated fatty acid response region in stearoyl-CoA desaturase gene 1. Biochim Biophys Acta. (1997) 1349:33–42. 10.1016/S0005-2760(97)00069-6 9421194

[20] Koeberle A Löser K Thürmer M Biochimica et biophysica acta Stearoyl-CoA desaturase-1 and adaptive stress signaling. BBA Mol Cell Biol Lipids. (2016) 1861:1719–26. 10.1016/j.bbalip.2016.08.009 27550503

[21] Gabbi C Warner M Gustafsson JÅ. Action mechanisms of liver X receptors. Biochem Biophys Res Commun. (2014) 446:647–50. 10.1016/j.bbrc.2013.11.077 24300092

[22] Huang C. Natural modulators of liver X receptors. J Integr Med. (2014) 12:76–85. 10.1016/S2095-4964(14)60013-3 24666673

[23] Oliveira AF Cunha DA Ladriere L Igoillo-Esteve M Bugliani M Marchetti P. *In vitro* use of free fatty acids bound to albumin: a comparison of protocols. Biotechniques. (2015) 58:228–33. 10.2144/000114285 25967901

[24] Baylin A Kabagambe EK Siles X Campos H. Adipose tissue biomarkers of fatty acid intake. Am J Clin Nutr. (2002) 76:750–7. 10.1093/ajcn/76.4.750 12324287

[25] Cnop M Hannaert JC Hoorens A Eizirik DL Pipeleers DG. Inverse relationship between cytotoxicity of free fatty acids in pancreatic islet cells and cellular triglyceride accumulation. Diabetes. (2001) 50:1771–7. 10.2337/diabetes.50.8.1771 11473037

[26] Audouard E Houben S Masaracchia C Yilmaz Z Suain V Authelet M. High-molecular-weight paired helical filaments from alzheimer brain induces seeding of wild-type mouse tau into an argyrophilic 4R tau pathology *in vivo*. Am J Pathol. (2016) 186:2709–22. 10.1016/j.ajpath.2016.06.008 27497324

[27] Louis C Van den Daelen C Tinant G Bourez S Thomé J-P Donnay I. Efficient *in vitro* adipocyte model of long-term lipolysis: a tool to study the behavior of lipophilic compounds. In Vitro Cell Dev Biol Anim. (2014) 50:507–18. 10.1007/s11626-014-9733-6 24477563

[28] Jamroz-wiśniewska A Wójcicka G. Horoszewicz K Liver X receptors (LXRs). Part I: Structure function, regulation of activity, role in lipid metabolism. Cześć II: Działania niezwiazane z gospodarka lipidowa, znaczenie w patologii i implikacje terapeutyczne. Postep Hig Med Dosw Online. (2007) 61:760–85. Available online at: https://www.google.com/url?sa=t&rct=j&q=&esrc=s&source=web&cd=7&ved=2ahUKEwiA0PvD-57lAhVKKlAKHaMbDcEQFjAGegQIARAC&url=https%3A%2F%2Fpdfs.semanticscholar.org%2Fd83f%2Fc6d7c612f0caba07fe36ac15280edf4dc6c6.pdf&usg=AOvVaw288uMAVMxbvbsTFm0Uk3NC

[29] Liu G Lynch JK Freeman J Liu B Xin Z Zhao H. Discovery of potent, selective, orally bioavailable stearoyl-CoA desaturase 1 inhibitors. J Med Chem. (2007) 50:3086–100. 10.1021/jm070219p 17530838

[30] Minville-Walz M Gresti J Pichon L Bellenger S Bellenger J Narce M. Distinct regulation of stearoyl-CoA desaturase 1 gene expression by cis and trans C18:1 fatty acids in human aortic smooth muscle cells. Genes Nutr. (2012) 7:209–16. 10.1007/s12263-011-0258-2 22057664 PMC3316751

[31] Sampath H Ntambi JM. The role of stearoyl-CoA desaturase in obesity, insulin resistance, and inflammation. Ann N Y Acad Sci. (2011) 1243:47–53. 10.1111/j.1749-6632.2011.06303.x 22211892

[32] Laffitte BA Chao LC Li J Walczak R Hummasti S Joseph SB. Activation of liver X receptor improves glucose tolerance through coordinate regulation of glucose metabolism in liver and adipose tissue. Proc Natl Acad Sci USA. (2003) 100:5419–24. 10.1073/pnas.0830671100 12697904 PMC154360

[33] Dalen KT Ulven SM Bamberg K Gustafsson J-Å Nebb HI. Expression of the insulin-responsive glucose transporter GLUT4 in adipocytes is dependent on liver X receptor α. J Biol Chem. (2003) 278:48283–91. 10.1074/jbc.M302287200 12970362

[34] Zelcer N Tontonoz P. Liver X receptors as integrators of metabolic and inflammatory signaling. J Clin Invest. (2006) 116:607–14. 10.1172/JCI27883 16511593 PMC1386115

[35] Kim W-K Meliton V Park KW Hong C Tontonoz P Niewiadomski P. Negative regulation of Hedgehog signaling by liver X receptors. Mol Endocrinol. (2009) 23:1532–43. 10.1210/me.2008-0453 19608643 PMC2754896

[36] Johnson JS Meliton V Kim WK Lee K-B Wang JC Nguyen K. Novel oxysterols have pro-osteogenic and anti-adipogenic effects *in vitro* and induce spinal fusion *in vivo*. J Cell Biochem. (2011) 112:1673–84. 10.1002/jcb.23082 21503957 PMC3080246

[37] Kim W-K Meliton V Tetradis S Weinmaster G Hahn TJ Carlson M. Osteogenic oxysterol, 20(S)-hydroxycholesterol, induces notch target gene expression in bone marrow stromal cells. J Bone Miner Res. (2010) 25:782–95. 10.1359/jbmr.091024 19839776 PMC3153332

[38] Matsushita K Morello F Zhang Z Masuda T Iwanaga S Steffensen KR. Nuclear hormone receptor LXRα inhibits adipocyte differentiation of mesenchymal stem cells with Wntbeta-catenin signaling. Lab Investig. (2016) 96:230–8. 10.1038/labinvest.2015.141 26595172 PMC4731266

[39] Kim H-J Yoon K-A Yoon H-J Hong JM Lee M-J Lee I-K. Liver X receptor activation inhibits osteoclastogenesis by suppressing NF-κB activity and c-Fos induction and prevents inflammatory bone loss in mice. J Leukoc Biol. (2013) 94:99–107. 10.1189/jlb.1112601 23657115

[40] Remen KMR Henning P Lerner UH Gustafsson J-Å Andersson G. Activation of liver X receptor (LXR) inhibits receptor activator of nuclear factor κB ligand (RANKL)-induced osteoclast differentiation in an LXRβ-dependent mechanism. J Biol Chem. (2011) 286:33084–94. 10.1074/jbc.M111.235937 21784849 PMC3190927

[41] Kleyer A Scholtysek C Bottesch E Hillienhof U Beyer C Distler JH. Liver X receptors orchestrate osteoblast/osteoclast crosstalk and counteract pathologic bone loss. J Bone Miner Res. (2012) 27:2442–51. 10.1002/jbmr.1702 22806960

[42] Alessio N Özcan S Tatsumi K Murat AAA Peluso G Dezawa M. The secretome of MUSE cells contains factors that may play a role in regulation of stemness, apoptosis and immunomodulation. Cell Cycle. (2017) 16:33–44. 10.1080/15384101.2016.1211215 27463232 PMC5270533

[43] Kohro T Nakajima T Wada Y Sugiyama A Ishii M Tsutsumi S. Genomic structure and mapping of human orphan receptor LXR alpha: upregulation of LXRa mRNA during monocyte to macrophage differentiation. J Atheroscler Thromb. (2000) 7:145–51. 10.5551/jat1994.7.145 11480455

[44] Schulman IG. Liver X receptors link lipid metabolism and inflammation. FEBS Lett. (2017) 591:2955–7. 10.1002/1873-3468.12702 28555747 PMC5638683

[45] Joseph SB Castrillo A Laffitte BA Mangelsdorf DJ Tontonoz P. Reciprocal regulation of inflammation and lipid metabolism by liver X receptors. Nat Med. (2003) 9:213–9. 10.1038/nm820 12524534

[46] Wang Y Li CC Cheng K Zhang RR Narsinh K Li S. Activation of liver X receptor improves viability of adipose-derived mesenchymal stem cells to attenuate myocardial ischemia injury through TLR4/NF-κB and Keap-1/Nrf-2 signaling pathways. Antioxid Redox Signal. (2014) 21:2543–57. 10.1089/ars.2013.5683 24915051 PMC4245883

[47] Castrillo A Joseph SB Vaidya SA Haberland M Fogelman AM Cheng G. Crosstalk between LXR and toll-like receptor signaling mediates bacterial and viral antagonism of cholesterol metabolism. Mol Cell. (2003) 12:805–16. 10.1016/S1097-2765(03)00384-8 14580333

[48] Peter A Weigert C Staiger H Machicao F Schick F Machann J. Individual stearoyl-CoA desaturase 1 expression modulates endoplasmic reticulum stress and inflammation in human myotubes and is associated with skeletal muscle lipid storage and insulin sensitivity *in vivo*. Diabetes. (2009) 58:1757–65. 10.2337/db09-0188 19478146 PMC2712792

[49] Matsui H Yokoyama T Sekiguchi K Iijima D Sunaga H Maniwa M. Stearoyl-Coa desaturase-1 (SCD1) augments saturated fatty acid-induced lipid accumulation and inhibits apoptosis in cardiac myocytes. PLoS ONE. (2012) 7:e33283. 10.1371/journal.pone.0033283 22413010 PMC3297642

[50] Bedi S Hines GV Lozada-Fernandez V V de Jesus Piva C Kaliappan A Rider SD. Fatty acid binding profile of the liver X receptor α. J Lipid Res. (2017) 58:393–402. 10.1194/jlr.M072447 28011707 PMC5282955

[51] Karaskov E Scott C Zhang L Teodoro T Ravazzola M Volchuk A Chronic palmitate but not oleate exposure induces endoplasmic reticulum stress, which may contribute to INS-1 pancreatic beta-cell apoptosis. Endocrinology. (2006) 147:3398–407. 10.1210/en.2005-1494 16601139

[52] Thörn K Hovsepyan M Bergsten P. Reduced levels of SCD1 accentuate palmitate-induced stress in insulin-producing β-cells. Lipids Health Dis. (2010) 9:108. 10.1186/1476-511X-9-108 20920255 PMC2955640

[53] Busch AK Gurisik E Cordery DV Sudlow M Denyer GS Ross Laybutt D. Increased fatty acid desaturation and enhanced expression of stearoyl coenzyme A desaturase protects pancreatic β-cells from lipoapoptosis. Diabetes. (2005) 54:2917–24. 10.2337/diabetes.54.10.2917 16186393

[54] Ariyama H Kono N Matsuda S Inoue T Arai H. Decrease in membrane phospholipid unsaturation induces unfolded protein response. J Biol Chem. (2010) 285:22027–35. 10.1074/jbc.M110.126870 20489212 PMC2903364

[55] Yen C-LE Stone SJ Koliwad S Harris C Farese RV. Thematic review series: glycerolipids. DGAT enzymes and triacylglycerol biosynthesis. J Lipid Res. (2008) 49:2283–301. 10.1194/jlr.R800018-JLR200 18757836 PMC3837458

[56] Chamulitrat W Liebisch G Xu W Gan-Schreier H Pathil A Schmitz G. Ursodeoxycholyl lysophosphatidylethanolamide inhibits lipoapoptosis by shifting fatty acid pools toward monosaturated and polyunsaturated fatty acids in mouse hepatocytes. Mol Pharmacol. (2013) 84:696–709. 10.1124/mol.113.088039 23974795

[57] Li Y Ge M Ciani L Kuriakose G Westover EJ Dura M. Enrichment of endoplasmic reticulum with cholesterol inhibits sarcoplasmic-endoplasmic reticulum calcium ATPase-2b activity in parallel with increased order of membrane lipids: implications for depletion of endoplasmic reticulum calcium stores and apoptos. J Biol Chem. (2004) 279:37030–9. 10.1074/jbc.M405195200 15215242

[58] Holzer RG Park E-J Li N Tran H Chen M Choi C. Saturated fatty acids induce c-Src clustering within membrane subdomains, leading to JNK activation. Cell. (2011) 147:173–84. 10.1016/j.cell.2011.08.034 21962514 PMC3295636

[59] Knecht V Mark AE Marrink S-J. Phase behavior of a phospholipid/fatty acid/water mixture studied in atomic detail. J Am Chem Soc. (2006) 128:2030–4. 10.1021/ja056619o 16464104

[60] Leekumjorn S Cho HJ Wu Y Wright NT Sum AK Chan C. The role of fatty acid unsaturation in minimizing biophysical changes on the structure and local effects of bilayer membranes. Biochim Biophys Acta Biomembr. (2009) 1788:1508–16. 10.1016/j.bbamem.2009.04.002 19371719 PMC2698950

[61] Bijelic R Balaban J Milicevic S. Correlation of the lipid profile, BMI and bone mineral density in postmenopausal women. Mater Socio Medica. (2016) 28:412. 10.5455/msm.2016.28.412-415 28144189 PMC5239653

[62] Liu F Wang W Yang L Wang B Wang J Chai W. An epidemiological study of etiology and clinical characteristics in patients with nontraumatic osteonecrosis of the femoral head. J Res Med Sci. (2017) 22:15. 10.4103/1735-1995.200273 28458706 PMC5367210

[63] Okazaki S Nagoya S Matsumoto H Mizuo K Sasaki M Watanabe S. Development of non-traumatic osteonecrosis of the femoral head requires toll-like receptor 7 and 9 stimulations and is boosted by repression on nuclear factor kappa B in rats. Lab Investig. (2015) 95:92–9. 10.1038/labinvest.2014.134 25384124 PMC7100527

[64] Han Y Si M Zhao Y Liu Y Cheng K Zhang Y. Progranulin protects against osteonecrosis of the femoral head by activating ERK1/2 pathway. Inflammation. (2017) 40:946–55. 10.1007/s10753-017-0539-z 28247166

[65] Rong X Albert CJ Hong C Duerr MA Chamberlain BT Tarling EJ. LXRs regulate ER stress and inflammation through dynamic modulation of membrane phospholipid composition. Cell Metab. (2013) 18:685–97. 10.1016/j.cmet.2013.10.002 24206663 PMC3889491

[66] Tangirala RK Bischoff ED Joseph SB Wagner BL Walczak R Laffitte BA. Identification of macrophage liver X receptors as inhibitors of atherosclerosis. Proc Natl Acad Sci. (2002) 99:11896–901. 10.1073/pnas.182199799 12193651 PMC129365

[67] Fernández-Veledo S Nieto-Vazquez I de Castro J Ramos MP Brüderlein S Möller P. Hyperinsulinemia induces insulin resistance on glucose and lipid metabolism in a human adipocytic cell line: paracrine interaction with myocytes. J Clin Endocrinol Metab. (2008) 93:2866–76. 10.1210/jc.2007-2472 18430774

[68] Gao M Bu L Ma Y Liu D. Concurrent activation of liver X receptor and peroxisome proliferator-activated receptor alpha exacerbates hepatic steatosis in high fat diet-induced obese mice. PLoS ONE. (2013) 8:e65641. 10.1371/journal.pone.0065641 23762402 PMC3676322

[69] Maqdasy S Trousson A Tauveron I Volle DH Baron S Lobaccaro J-MA. Once and for all, LXRα and LXRβ are gatekeepers of the endocrine system. Mol Aspects Med. (2016) 49:31–46. 10.1016/j.mam.2016.04.001 27091047

[70] Liang W Wu X Fang W Zhao Y Yang Y Hu Z. Network meta-analysis of erlotinib, gefitinib, afatinib and icotinib in patients with advanced non-small-cell lung cancer harboring EGFR mutations. PLoS ONE. (2014) 9:e85245. 10.1371/journal.pone.0085245 24533047 PMC3922700

[71] Zhang J Song F Zhao X Jiang H Wu X Wang B. EGFR modulates monounsaturated fatty acid synthesis through phosphorylation of SCD1 in lung cancer. Mol Cancer. (2017) 16:127. 10.1186/s12943-017-0704-x 28724430 PMC5518108

[72] Lu S Xu F Hu W Niu Z Cai H Chen Y. SCD1 methylation in subcutaneous adipose tissue associated with menopausal age. Climacteric. (2019) 22:395–402. 10.1080/13697137.2019.1571028 30777456

[73] Li X Shet K Xu K Rodríguez JP Pino AM Kurhanewicz J Unsaturation level decreased in bone marrow fat of postmenopausal women with low bone density using high resolution magic angle spinning (HRMAS) 1H NMR spectroscopy. Bone. (2017) 105:87–92. 10.1016/j.bone.2017.08.014 28823880 PMC5650928

