# Supplementary material for: Induction of Stearoyl-CoA 9-Desaturase 1 Protects Human Mesenchymal Stromal Cells Against Palmitic Acid-Induced Lipotoxicity and Inflammation
Source: Front Endocrinol (Lausanne). 2019 Oct 24;10:726. doi: 10.3389/fendo.2019.00726 (PMC6822410; doi:10.3389/fendo.2019.00726)
Supplement: Supplementary file 1 [file Data_Sheet_1.docx]

**
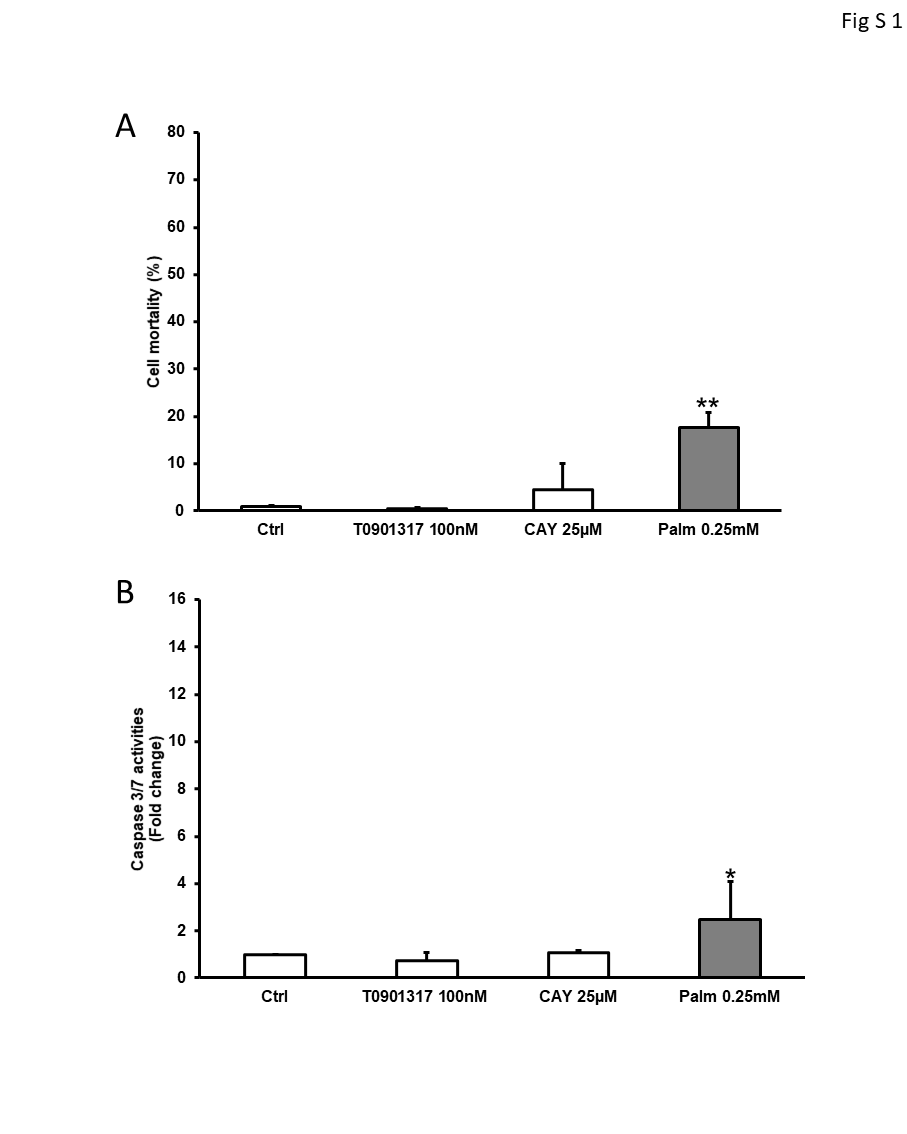
**

**Supplemental Fig.1: CAY sand T0901317 have no signifanct effect on cell viability and caspases 3/7 activation.**

hMSC were pretreated during 16h with T0901317 and treated 48h with T0901317, CAY 25µM in the absence (white column) or presence of Palm 0.25 mM (grey column). Cell death **(A)** was quantified by nuclear staining with Hoechst and propidium iodide. Values are mean ± SEM of 4 individual experiments. Caspases-3/7 activity **(B)** was measured using the Caspases-3/7 Glo assay. Values are expressed relative to Ctrl and are mean ± SEM of 3 individual experiments. * p< 0.05; ** p< 0.01 vs Ctrl; # p< 0.05; ## p< 0.01 vs Palm 0.25 mM; $ p< 0.05; $$ p< 0.01 vs Palm 0.25 mM+T0901317 100 nM.

**
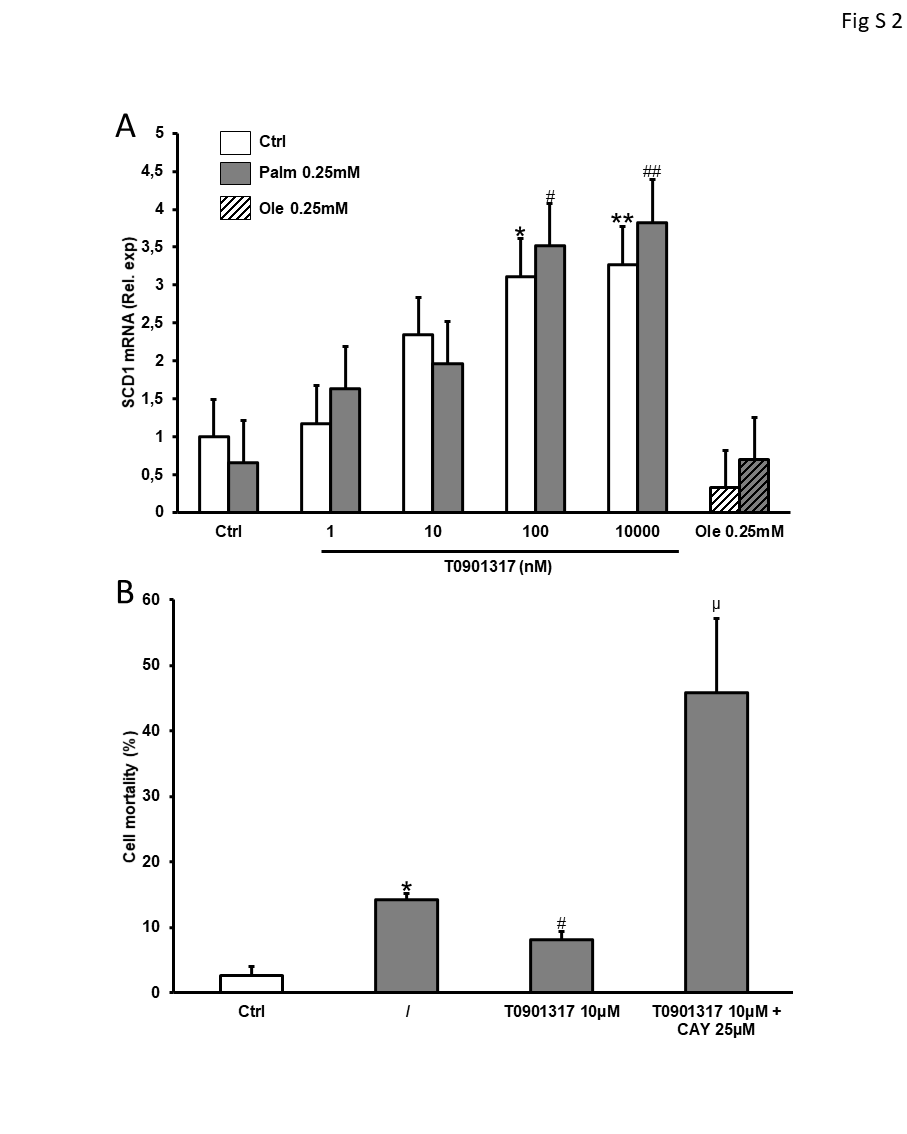
Supplemental Fig.2: T0901317 increases SCD1 expression in SaOS-2 cells**. SaOS-2 cells were pretreated during 24h with T0901317 and treated 16h with T0901317, CAY 25 µM, Ole 0.25 mM (shaded column) in absence (white column), or in the presence of Palm 0.25 mM (grey column). SCD1 **(A)** expression was quantified by qPCR using the ΔΔCT method. Values were normalized for HPRT1 expression and are expressed as the ΔΔCT compared to control (Ctrl). Results are means ± SEM of 3-5 individual experiments. Cell death **(B)** was quantified by nuclear staining with Hoechst and propidium iodide. Values are mean ± SEM of 3-4 individual experiments. * p<0.05 vs Ctrl; # p<0.05 vs Palm 0.25 mM; µ p< 0.05 vs Palm 0.25mM + T0901317 10µM.


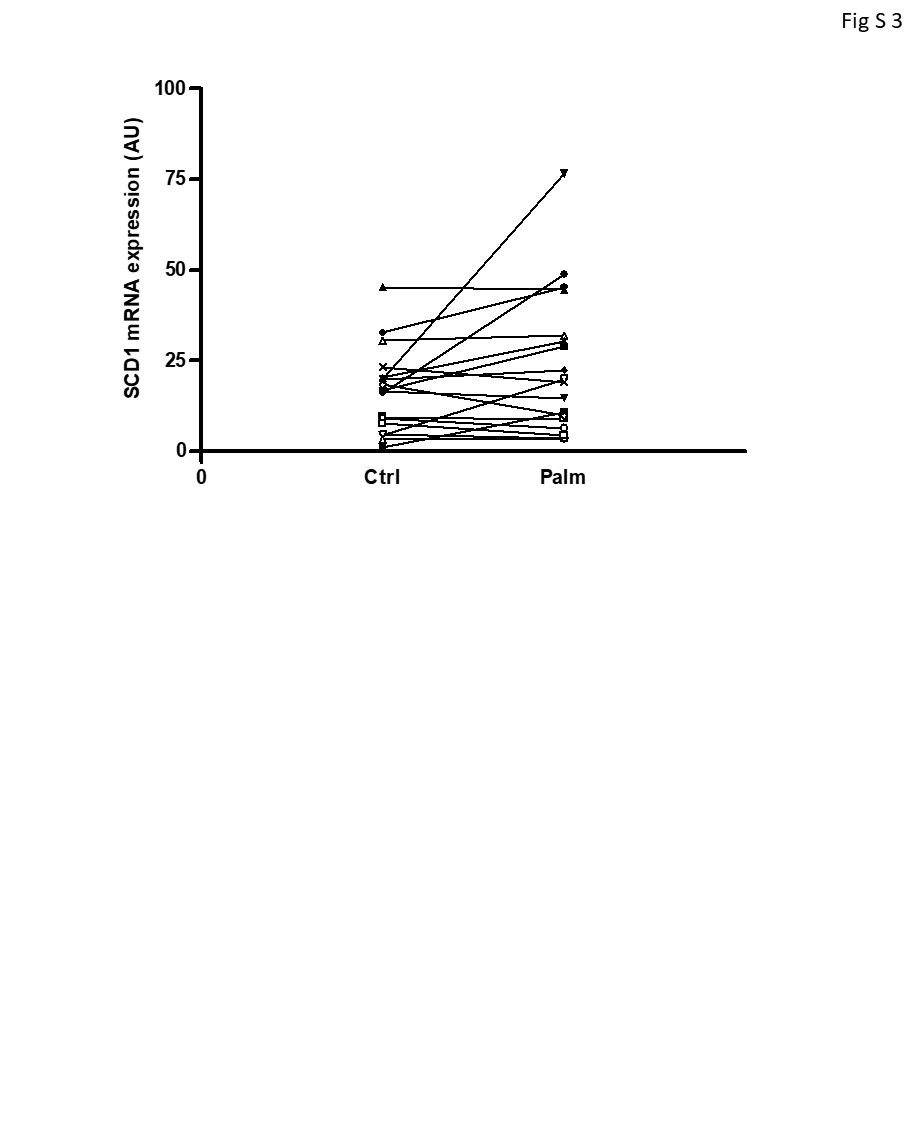


**Supplemental Fig.3: SCD1 mRNA expression is no significantly increased by Palm**. Change in SCD1 mRNA expression expressed in arbitrary unit (AU) in hMSCs of 18 healthy volunteer treated 24h with or without 0.25 mM Palm. Palm tended to increased SCD1 mRNA expression by 1.3 but not in a significant manner.
